# Development and Validation of a nomogram for forecasting survival of alcohol related hepatocellular carcinoma patients

**DOI:** 10.3389/fonc.2022.976445

**Published:** 2022-11-11

**Authors:** Tao Yan, Chenyang Huang, Jin Lei, Qian Guo, Guodong Su, Tong Wu, Xueyuan Jin, Caiyun Peng, Jiamin Cheng, Linzhi Zhang, Zherui Liu, Terence Kin, Fan Ying, Suthat Liangpunsakul, Yinyin Li, Yinying Lu

**Affiliations:** ^1^ Comprehensive Liver Cancer Center, The Fifth Medical Center of PLA General Hospital, Beijing, China; ^2^ The Second School of Clinical Medicine, Southern Medical University, Guangzhou, China; ^3^ The First Affiliated Hospital, Guizhou Medical University, Guiyang, China; ^4^ Medical Quality Control Department, The Fifth Medical Center of PLA General Hospital, Beijing, China; ^5^ Department of Applied Biology and Chemical Technology, The Hong Kong Polytechnic University, Hong Kong, Hong Kong SAR, China; ^6^ Division of Gastroenterology and Hepatology, Department of Medicine, Indiana University School of Medicine, Indianapolis, IN, United States; ^7^ Center for Synthetic and Systems Biology (CSSB), Tsinghua University, Beijing, China

**Keywords:** hepatocellular carcinoma, nomogram, prognosis, overall survival, alcohol

## Abstract

**Background:**

With the increasing incidence and prevalence of alcoholic liver disease, alcohol-related hepatocellular carcinoma has become a serious public health problem worthy of attention in China. However, there is currently no prognostic prediction model for alcohol-related hepatocellular carcinoma.

**Methods:**

The retrospective analysis research of alcohol related hepatocellular carcinoma patients was conducted from January 2010 to December 2014. Independent prognostic factors of alcohol related hepatocellular carcinoma were identified by Lasso regression and multivariate COX proportional model analysis, and the nomogram model was constructed. The reliability and accuracy of the model were assessed using the concordance index(C-Index), receiver operating characteristic (ROC) curve and calibration curve. Evaluate the clinical benefit and application value of the model through clinical decision curve analysis (DCA). The prognosis was assessed by the Kaplan-Meier (KM) survival curve.

**Results:**

In sum, 383 patients were included in our study. Patients were stochastically assigned to training cohort (n=271) and validation cohort (n=112) according to 7:3 ratio. The predictors included in the nomogram were splenectomy, platelet count (PLT), creatinine (CRE), Prealbumin (PA), mean erythrocyte hemoglobin concentration (MCHC), red blood cell distribution width (RDW) and TNM. Our nomogram demonstrated excellent discriminatory power (*C*-index) and good calibration at 1-year, 3-year and 5- year overall survival (OS). Compared to TNM and Child-Pugh model, the nomogram had better discriminative ability and higher accuracy. DCA showed high clinical benefit and application value of the model.

**Conclusion:**

The nomogram model we established can precisely forcasting the prognosis of alcohol related hepatocellular carcinoma patients, which would be helpful for the early warning of alcohol related hepatocellular carcinoma and predict prognosis in patients with alcoholic hepatocellular carcinoma.

## Introduction

Hepatocellular carcinoma (HCC), a major public health burden worldwide, is one of the leading reasons of cancer-related mortality and there were an estimated 854,000 incident HCC cases (75% increase from 1990) and 810,000 cancer-related deaths worldwide in 2015 (1, 2). The most common risk factor for HCC is cirrhosis from various etiologies. Incidence and mortality ratio of liver cancer have decreased in numerous high-risk level countries due to the decline in the incidence of hepatitis B and hepatitis C infection (3). Other non-viral etiologies, excessive consumption of alcohol and nonalcoholic fatty liver disease, are now major risk factors for HCC (4, 5). The rate of alcohol use disorder and alcohol-associated liver disease is on the rise resulting in an increase in the incidence of alcohol-associated cirrhosis (6). A modeling study forecasts a significant increase in the incidence of alcohol-associated hepatocellular carcinoma (alcohol related hepatocellular carcinoma) in the next decade (7). An epidemiology study demonstrated that excessive alcohol consumption more than 80 grams daily for over a 10-year period significantly increases the risk of alcohol related hepatocellular carcinoma by 5-fold (8, 9).

A nomogram is a statistical model being used as an algorithm to maximize the predictive accuracy of a given outcome, i.e., overall survival at the individual level in a patient with cancer (10, 11). A study reported a nomogram prediction of individual prognosis of patients with HCC, primarily from viral hepatitis B, with good accuracy (12). Others have reported the nomogram for forcasting survival in early stage HCC, end stage HCC after hepatic arterial infusion chemotherapy, HCC with portal vein tumor thrombus after resection, and recurrence of HCC after surgery (13–17). Given an increase in the incidence of alcohol related hepatocellular carcinoma and the report that patients with alcohol related hepatocellular carcinoma had a markedly reduced overall survival, mainly because of a worse liver function and tumor characteristics at diagnosis, when compared to patients with non–alcohol-related HCC, construction of a nomogram with good predictive accuracy of patients with alcohol related hepatocellular carcinoma, is needed to tailor the treatment plans at the individual level. The primary purpose of our research was to develop and verification a nomogram for predicting survival in patients with alcohol related hepatocellular carcinoma.

## Methods

### Study cohort and design

**Figure 1:**
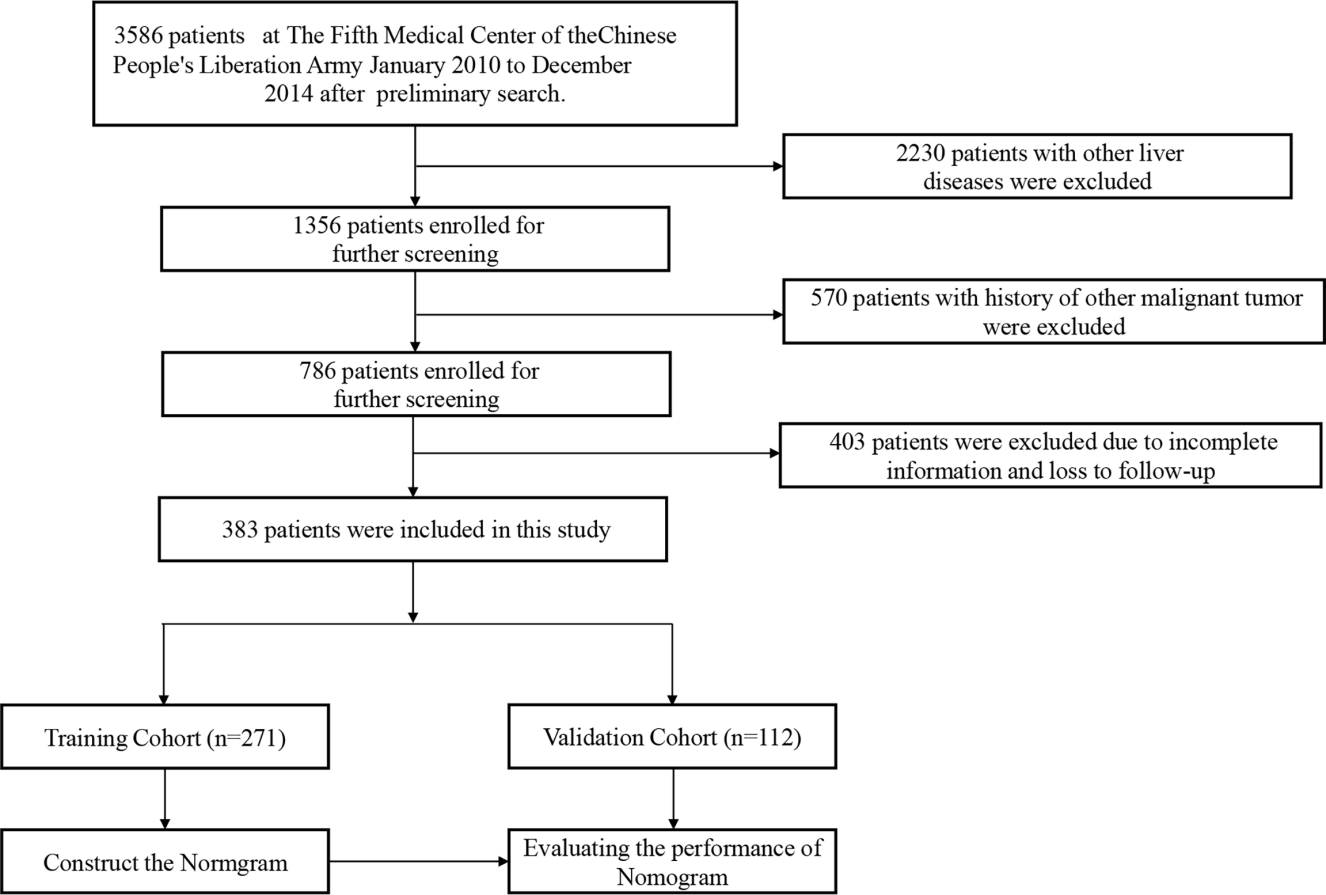
Flow gram of the patients selection.

We conducted a retrospective cohort study of patients seen at the comprehensive liver cancer center, the 5^th^ medical center of PLA General Hospital between January 2010 and December 2014. The followings were inclusion criteria: (1) history of hazardous alcohol consumption, as defined by daily alcohol intake >80 g for men and 60 g for women, for more than 10 years; (2) histopathological diagnosis of HCC; (3) no other known causes of underlying liver disease, and (4) aged between 18-80 years old. The exclusion criteria were as follows: (1) patients with other concurrent chronic liver diseases, such as those with serum positivity to hepatitis B and C virus, autoimmune hepatitis, drug-induced hepatitis, hemochromatosis, and primary biliary cirrhosis; (2) patients with history of other malignancies, (3) history of liver transplantation surgery, (4) incomplete clinical data, and (5) history of HIV infection. (6) death from non-neoplastic causes. The end point of follow-up was death or last follow up. The patients selection process is illustrated in **Figure 1**. By entering the diagnose keywords “primary hepatocellular carcinoma with alcoholic cirrhosis” in the electronic medical record system, we retrieved 3586 patients. Then we identified 383 patients who fulfilled the inclusion and exclusion criteria. They were stochastically dichotomized into a training cohort (n=271) and internal validation cohort (n=112) in a 7:3 ratio. The Institutional Ethics Committee approved the study.

### Baseline clinical and laboratory data collection

The following baseline data were collected: (1) baseline demographics and body mass index; (2) presence and absence of other co-morbidities such as diabetes and hypertension; (3) alcohol consumption data, types of alcohol beverages and the average daily alcohol consumption; (4) history of upper gastrointestinal bleeding; (5) surgical history, such as splenectomy; (6) complications of portal hypertension, such as hepatic encephalopathy and ascites, (7) history of HCC treatment, (8) laboratory data; (9) baseline Child-Pugh classification; and (10) tumor staging at baseline using TNM systems (18).

### Follow up period

Patients received regular follow up every 3-6 months. At each visit, physical examination, routine laboratory tests, serum alpha-fetoprotein (AFP), other tumor markers and radiographic imaging, either with computed tomography (CT) or magnetic resonance imaging (MRI) were performed. The treatment algorithm for HCC after the diagnosis was based on the National Comprehensive Cancer Network guideline (19). The treatment modalities during the follow up period were noted including liver resection, radiofrequency ablation, percutaneous ethanol injection, radio-interventional therapy, systemic therapy and liver transplantation.

### Statistical analysis

The primary endpoint of the study was the overall survival (OS), defined as the time from the first diagnosis to death from any cause, until the last known follow-up or the study closure date of December 31, 2018. Basic descriptive statistics, including mean, standard deviations (SD), and frequencies (percentages) were used to characterize the dataset in both training and validation cohorts. Categorical data were compared using Person χ 2 test or Fisher’s exact test and continuous variables were compared using Student’s t test or Mann-whitey U test. LASSO regression (20) analyses were used for variable selection and shrinkage in multivariate Cox proportional hazards model for factors independently associated with the overall survival in alcohol related hepatocellular carcinoma patients (21). Kaplan-Meier curves for selected variables were created in a stepwise manner. We used the training cohort to generate nomogram based on the multivariate regression to predict 1- year, 3-year and 5- years OS, using the package of “rms”. Web calculator was built by the “shiny” package. The area under the curve (AUC) and Harrell’s concordance index (C-index) (22) were used to evaluate the consistency and accuracy of the prediction model, while calibration was performed by observing the survival probability with Kaplan-Meier estimating in both training and validation cohorts. Decision curve analysis (DCA) (23), a method for evaluating the diagnostic accuracy of a diagnostic model, was conducted by assuming that the threshold probability of a disease or event at which a patient would opt for treatment is informative of how the patient weighs the relative harms of a false-positive and a false-negative prediction (23). The relationship was used to derive the net benefit of the model across different threshold probabilities. The DCA was generated by plotting net benefit against threshold probability. All statistical analyses were performed by using R (ver. 4.1.2; R Foundation for Statistical Computing, Vienna, Austria). P value < 0.05 was considered to have statistical significance. The online calculator was constructed using the Shiny package for R.

During the study period, a total of 3,586 patients records were reviewed through the electronic medical record system. Following the exclusion criteria, 383 patients were eventually included in our study cohort. Of these, a total of 271 and 112 patients were stochastically assigned to the training and validation cohorts, respectively. The baseline demographics and laboratory data of both cohorts are shown in **Table 1**. There was no significant difference in age (66.36 years vs. 67.03 years, p=0.58), body mass index (24.46 kg/m^2^ vs. 24.54 kg/m^2^, p=0.98), average alcohol consumption per day (386.72 ml vs. 371.65 ml, p=0.65), and average duration of alcohol consumption (27.46 years vs. 27.07 years, p=0.65) between both groups. The baseline performance status (p=0.18), Child-Pugh classification (p=0.59), and TNM tumor staging (p=0.92) between training and validation cohorts were also comparable. The baseline laboratory data were also comparable, except for the serum glucose (5.76 mmol/L vs. 6.32 mmol/L, p=0.02) and prothrombin time (13 seconds vs. 13.55 seconds, p=0.01).

**Table 1:** Baseline characteristics of training and validation cohorts after deleting unknown/missing data.

Variable	Training cohort (N = 271)	Validation cohort (N = 112)	P value
Demographics and medical history			
Age, y	66.37 (9.74)	67.03 (9.81)	0.58
Body Mass Index	24.46 (3.67)	24.54 (3.75)	0.98
Hypertension, no/yes	208/63 (76.75/23.25)	84/28 (75.00/25/00)	0.71
Types of alcohol			0.27
Liquor(53°)	138 (50.92%)	50 (44.64%)	
Wine(15°)	1 (0.36%)	2 (1.79%)	
Beer(3°)	2 (0.74%)	1 (0.36%)	
Liquor but unknow degrees	130 (47.97%)	59 (52.68%)	
Acohol consumption per day(ml)	386.72 (309.52)	371.65 (239.73)	0.65
Alcohol consumption duration time(years)	27.46 (10.38)	27.07 (11.28)	0.65
Upper alimentary canal bleeding history, no/yes	230/41(84.87/15.13)	101/11 (90.18/9.82)	0.17
Tumour rupture bleeding history, no/yes	270/1 (99.64/0.36)	111/1 (99.64/0.36)	0.5
Hepatic encephalopathy			0.62
I/II	265 (97.79%)	108 (96.43%)	
III/IV	5 (1.84%)	4 (3.57%)	
No	1 (0.36%)	0 (0.00%)	
Ascites, no/yes	177/94 (65.31/34.69)	66/46 (58.93/41.07)	0.24
Fibrosis 4 Score	6.13 (5.93)	5.75 (5.14)	0.99
APRI	1.93 (4.31)	1.87 (3.36)	0.86
Laboratory parameters			
Glu, *mmol/L*	5.76 (2.11)	6.32 (2.60)	0.02
ALB, *g/L*	33.62 (6.12)	32.76 (6.16)	0.13
PA, *g/L*	116.99 (63.06)	107.68 (67.96)	0.075
DBIL, *μmol/L*	26.43 (47.50)	31.42 (62.33)	0.49
BIL, *μmol/L*	41.67 (59.63)	49.05 (77.88)	0.45
ALT, *U/L*	54.44 (138.57)	50.57 (63.89)	0.25
AST, *U/L*	84.49 (169.53)	74.01 (84.22)	0.85
ALP, *U/L*	197.55 (158.91)	220.60 (195.77)	0.65
GGT, *U/L*	240.68 (241.37)	243.93 (281.47)	0.2
TBA, *μmol/L*	38.29 (42.69)	48.39 (60.32)	0.3
CHE, *U/L*	4047.63 (1971.56)	3798.57 (1940.11)	0.21
LDH, *U/L*	262.80 (258.49)	248.58 (176.84)	0.58
ADA, *U/L*	23.93 (10.20)	25.38 (11.38)	0.26
AMY, *U/L*	59.75 (43.19)	57.21 (31.70)	0.6
CK, *U/L*	85.01 (82.34)	76.26 (48.61)	0.91
BUN, *mmol/L*	5.84 (3.61)	6.04 (4.38)	0.97
CRE, *umol/L*	83.84 (34.45)	82.90 (33.54)	0.36
UA, umol/L	323.93(124.89)	317.08 (124.65)	0.53
Ca, *mmol/L*	2.18 (0.20)	2.18 (0.20)	0.51
P, *mmol/L*	1.14 (0.25)	1.07 (0.25)	0.007
Mg, *mmol/L*	0.84 (0.12)	0.84 (0.16)	0.2
TC, *mmol/L*	3.75 (1.26)	3.61 (1.83)	0.032
TG, *mmol/L*	1.14 (0.67)	1.13 (0.70)	0.41
PT, *mg/dL*	13.00 (2.19)	13.55 (2.36)	0.01
PTA, *%*	78.94 (17.46)	73.96 (17.71)	0.009
INR	1.13 (0.17)	1.20 (0.35)	0.013
WBC, *10^9^/L*	5.96 (2.94)	6.03 (3.15)	0.89
RBC, *10^12^/L*	3.90 (0.76)	3.87 (0.78)	0.72
HGB, *g/L*	122.67 (24.63)	121.74 (26.50)	0.79
HCT, %	36.71 (7.60)	37.06 (9.58)	0.91
MCV, *fl*	94.99 (16.75)	96.36 (25.00)	0.89
MCHC, *g/L*	329.30 (44.17)	333.39 (31.52)	0.38
RDW, *%*	14.86 (2.84)	15.00 (2.88)	0.38
PLT, *10^9^/L*	148.03 (95.99)	140.63 (91.07)	0.49
Scoring & Grade system			
PST			0.18
0	144 (53.14%)	60 (53.57%)	
1	48 (17.71%)	14 (12.50%)	
2	70 (25.83%)	30 (26.79%)	
3	5 (1.85%)	7 (6.25%)	
4	4 (1.48%)	1 (0.89%)	
Child-Pugh			0.59
A	111 (40.96%)	46 (41.07%)	
B	124 (45.76%)	48 (42.86%)	
C	36 (13.28%)	18 (16.07%)	
MELD	54.88 (5.38)	55.98 (5.88)	0.47
TNM			0.92
I	88 (32.59%)	38 (34.86%)	
II	54 (20.00%)	21 (19.27%)	
IIIA	36 (13.33%)	11 (10.09%)	
IIIB	49 (18.15%)	18 (16.51%)	
IIIC	3(0.78%)	3 (0.78%)	
IVA	16 (5.93%)	9 (8.26%)	
IVB	26 (9.63%)	12 (11.01%)	

Values are presented as the median [standard deviation] or n (%).

Glu, glucose; ALB, albumin; PA, prealbumin; DBIL, direct bilirubin; BIL, bilirubin, ALT, alanine aminotransferase; AST, aspartate aminotransferase; ALP; U/L; GGT, glutamyltransferase; TBA, bile acid; CHE, cholinesterase; LDH, lactate dehydrogenase; ADA, adenosine deaminase; AMY, amylase; CK, Creatine Kinase; BUN, blood urea nitrogen; CRE, Creatinine; UA, uric acid; Ca, serum calcium; P, serum phosphorus; Mg, serum magnesium; TC, total cholesterol; TG, triglycerides; PT, prothrombin; PTA, prothrombin activity; INR, International normalized ratio of prothrombin; WBC, white blood cell; RBC, red blood cell count; HGB, hemoglobin; HCT, hematocrit; MCV, mean red blood cell volume; MCHC, mean hemoglobin concentration; RDW, red blood cell distribution width; PLT, platelet count; PST, performance status test; TNM, Tumor Node Metastasis.

A total of 304 (79.4%) patients died during the follow-up with the median overall survival of 21 months (95% CI 18.8–26.2). showed No significant difference in the survival curve between the training cohort and the validation cohort (**Figure S1**). The mean follow-up times were 35.0 and 37.0 months in the training and validation cohorts, respectively. The detailed treatment modalities of these patients were shown in **Supplementary Table 1**.

### Selection of variables associated with mortality and nomogram construction

**Figure 2:**
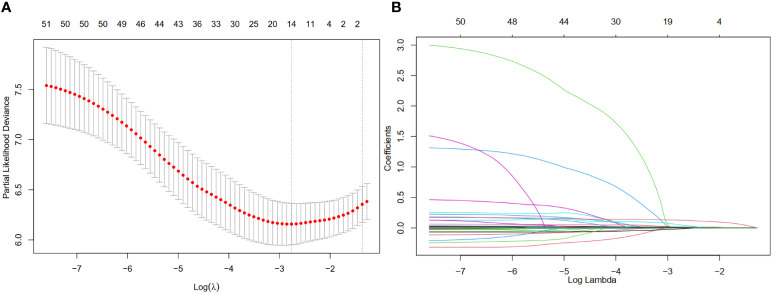
Feature selection using a least absolute shrinkage and selection operator (LASSO) regression model. **(A)** Tuning parameter (λ) selection for the LASSO model involved fivefold cross-validation using the minimal criteria. Dotted vertical lines were drawn at the optimal values; one standard error was added to each criterion to yield the 1-SE criteria. **(B)** The LASSO coefficients of the 49 features.

**Figure 3:**
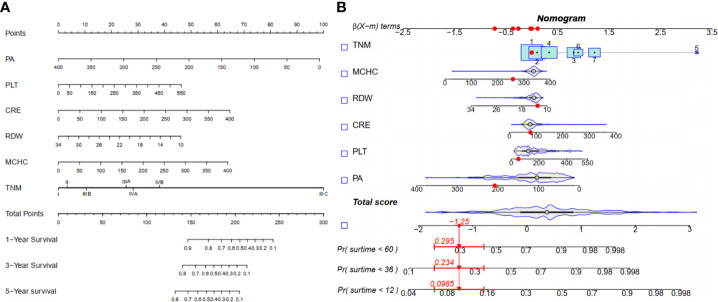
Nomogram for 1-, 3-, and 5-yearOS in alcohol related hepatocellular carcinoma patients. **(A)** Line segment static nomogram;**(B)** Line segment dynamic nomogram.

The LASSO coefficient profiles of 14 variables associated with mortality in patients with alcohol related hepatocellular carcinoma and cross-validation for parameter selection in the LASSO model were shown in **Figure 2**.** A** LASSO regression analysis was first conducted in the training cohort to identify variables associated with mortality in patients with alcohol related hepatocellular carcinoma. The following variables were included, upper alimentary canal bleeding history, serum pre-albumin (PA), serum bilirubin (BIL), gamma-glutamyl transferase (GGT), lactate dehydrogenase (LDH), adenosine deaminase (ADA), creatine kinase (CK), creatinine (CRE), white blood cells (WBC), mean red blood cell volume (MCV), total cholesterol (TC), mean corpuscular hemoglobin concentration (MCHC), platelet counts (PLT), red blood cell distribution width (RDW), Child-Pugh score, tumor lymph node metastasis staging (TNM) (**Figure 2**). The coefficient profiles of these 14 variables were included in the multivariate COX regression model. The following variables were independent predictors of mortality outcomes for alcohol related hepatocellular carcinoma patients in our study cohort, PA (Hazard ratio, HR 0.99, p<0.01), CRE (HR 1.01, p=0.03), MCHC (HR 1.01, p=0.04), RDW (HR 0.92, p=0.03), PLT (HR 1.01, p=0.02), TNM staging (**Table 2**). These variables were used to construct a line segment static and dynamic nomograms, as shown in **Figure 3**. Furthermore, in order to provide researchers with more convenient use, a online calculator for the nomogram prediction model had been constructed (https://chenyanghuang.shinyapps.io/DynNomogram/). Using the normal cut-off values based on our laboratory reference, we conducted Kaplan–Meier survival curve stratified by a respective cut-off value for each variable. We found that the baseline level of serum PA (cut-off value 170 g/L), PLT (cut-off value 100x10^9^ cells/L), and TNM staging were associated with survival outcome during follow up (**Supplementary Figures 2A–E**).

**Table 2:** Multivariate Cox regression analyses of variables.

Variables	HR	95%Cl (lower)	95%Cl (upper)	P
PA	0.99	0.98	1.00	**<0.01**
BIL	1.00	0.99	1.01	0.06
GGT	1.00	1.00	1.00	0.14
LDH	1.00	1.00	1.00	0.30
ADA	0.98	0.97	1.01	0.11
TC	1.05	0.93	1.18	0.46
CRE	1.01	1.00	1.01	**0.03**
WBC	0.98	0.92	1.05	0.62
MCV	0.99	0.98	1.01	0.31
MCHC	1.01	1.00	1.01	**0.04**
RDW	0.92	0.86	1.00	**0.03**
PLT	1.01	1.00	1.01	**0.02**
Child-Pluge				
A	ref	ref	\	\
B	1.09	0.70	1.71	0.70
C	1.63	0.44	6.09	0.47
TNM				
I	ref	ref	\	\
II	1.16	0.75	1.78	0.50
IIIA	2.09	1.30	3.36	**<0.01**
IIIB	1.37	0.87	2.15	0.17
IIIC	13.55	2.09	87.87	**<0.01**
IVA	2.34	1.28	4.28	**<0.01**
IVB	2.87	1.68	4.91	**<0.01**

Values: representative clinical indicators; HR, Hazard Ratio; 95%Cl (lower), 95% lower confidence interval; 95%Cl (upper), 95%upper confidence interval; PA, prealbumin; BIL, bilirubin; GGT, glutamyltransferase; LDH, lactate dehydrogenase; ADA, adenosine deaminase; TC, total cholesterol; CRE, Creatinine; WBC, white blood cell; MCV, mean red blood cell volume; MCHC, mean hemoglobin concentration; RDW, red blood cell distribution width; PLT, platelet count; TNM, Tumor Node Metastasis.

### ROC Curve and C-Index in the training and validation cohorts

**Figure 4:**
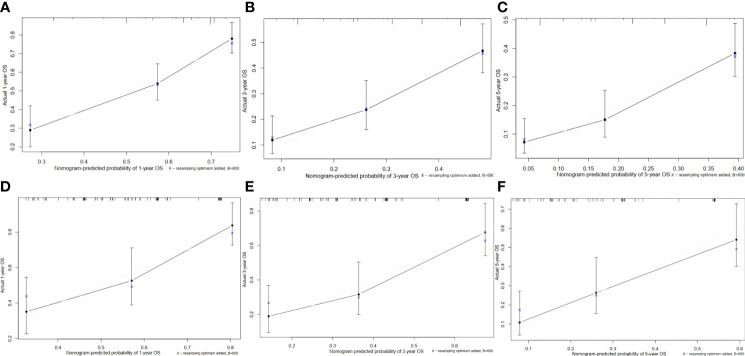
Calibration curves of the prognostic nomogram for each cohort. **(A)** Calibration curve of the nomogram for the training cohort at 1 year. **(B)** Calibration curve of the nomogram for the training cohort at 3 years. **(C)** Calibration curve of the nomogram for the training cohort at 5 years. **(D)** Calibration curve of the nomogram for the validation cohort at 1 year. **(E)** Calibration curve of the nomogram for the validation cohort at 3 years. **(F)** Calibration curve of the nomogram for the validation cohort at 5 years.

In the training cohort, the *C*-index of the nomogram for the overall survival prediction was 0.67 and the calibration plots for 1- year, 3-year and 5-year survival probabilities of patients showed excellent consistency between the forcast and actual survival (**Figures 4A–C**). In the validation cohort, the *C*-index of the nomogram for the overall survival prediction was 0.68; the calibration plots for the 1- year, 3-year and 5-year survival probabilities also showed an excellent consistency between the forcast and observed survival (**Figures 4D, E**).

**Figure 5:**
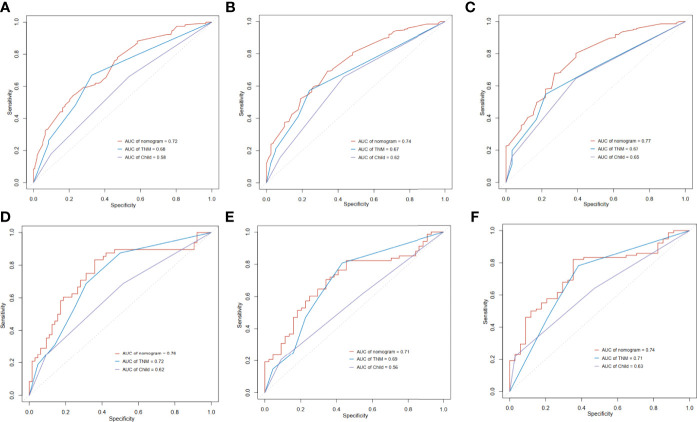
Time-dependent receiver operating characteristic (ROC) curves of the nomogram score, model based on TNM stage and model based on Child-Pugh. **(A)** 1 year in training cohort; **(B)** 3 years in training cohort; **(C)** 5 years in training cohort; **(D)** 1 year in validation cohort; **(E)** 3 years in validation cohort; **(F)** 5 years in validation cohort.

We next compared the prognostic ability of our nomogram to that of TNM and Child-Pugh score. In the training cohort, the 1-year area under the curves (AUCs) of the nomogram and the prognostic model based on TNM and Child-Pugh were 0.72, 0.68, and 0.58, respectively (**Figure 5A**). The 3-year AUCs of the nomogram, TNM, and Child-Pugh were 0.74, 0.67, and 0.62 respectively (**Figure 5B**), and the 5-year AUCs for each prognostic values were 0.77, 0.67, and 0.65 respectively (**Figure 5C**). In the validation cohort, the 1-year area under the curves (AUCs) of the nomogram and the prognostic model based on TNM and Child-Pugh were 0.76, 0.72, and 0.62, respectively (**Figure 5D**). The 3-year AUCs of the nomogram, TNM, and Child-Pugh were 0.71, 0.69, 0.56, respectively (**Figure 5E**), and the 5-year AUCs for each prognostic values were 0.74, 0.71, 0.63, respectively (**Figure 5F**).

### Decision curve analysis (DCA) for clinical utility of the nomogram

**Figure 6:**
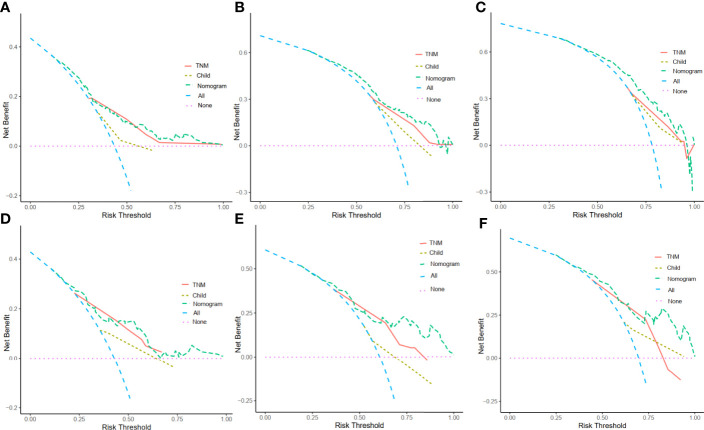
Decision curve analysis (DCA) for the nomogram. The y-axis represents net benefits, calculated by subtracting the relative harms (true positives) from the benefits (false positives). The x-axis measures the threshold probability. **(A)** DCA in the training cohort at 1 year. **(B)**DCA in the training cohort at 3 years. **(C)** DCA in the training cohort at 5 years. **(D)** DCAin the validation cohort at 1 year. **(E)** DCA in the validation cohort at 3 years. **(F)** DCA in the validation cohort at 5 years.

DCA enables the integration of patient or decision maker preferences into the analysis. The DCA of our nomogram and other prognostic variables, TNM and Child-Pugh score, in the training cohort and validation cohorts for 1-year, 3-year and 5-year survival were illustrated in **Figure 6**. The x-axis represented the risk threshold while the y-axis was the net benefit. We found that our nomogram demonstrated more net benefit than other prognostic variables in predicting the 1-year, 3-year and 5-year survival (**Figure 6**).

## Discussion

Globally, an estimated 741 300 of all new cases of cancer in 2020 were attributable to alcohol consumption and highest in eastern Asia (5·7%:3·6-7·9). One of the major risk factor for HCC development is underlying cirrhosis. While the incidence of HCC secondary to HBV and HCV infection is decreasing, the cases of HCC secondary to underlying alcohol-associated liver disease are on the rise (3). Several advancements have been made in the treatment options for HCC over the past few years, with different therapeutic modalities for different stages of the disease. Treatment decisions in general are individualized depending on patient overall performance status, the underlying liver function, and the disease stage (24). Surgical resection, local ablative therapies such as radiofrequency ablation, and liver transplantation offer potential cure for patients with underlying cirrhosis with the disease detected at an early stage in carefully selected patients (25). For intermediate-stage HCC, transarterial chemoembolization is the mainstay of treatment and a systemic therapy is the option for a selected group of patients with advanced disease. As with other cancers, HCC staging is essential for guiding the appropriate interventions and providing the overall prognosis, an important factor in treatment planning and decision making for both providers and patients. There are several staging systems to determine prognosis and predict the survival in patients with HCC; among them are Tumor, Node, Metastasis (TNM) staging and Barcelona Clinic Liver Cancer (BCLC) staging (26). Each staging system has its pros and cons. The TNM classification evaluates the extent of the primary tumor, the presence/absence of lymph node involvement, and/or extrahepatic metastasis (26). However, it does not take the liver functional status into account (26). The BCLC system consists of four elements, tumor extension, patient’s physical status, liver functional reserve, and cancer-related symptoms; it also provides guidance on treatment recommendations for each HCC stage (26). Both systems categorize patients collectively into each HCC stage in predicting the outcomes. They lack the granularity to determine the overall survival at the individual level. And there was no study on the prognosis of alcohol related hepatocellular carcinoma patients, so we do the first to study the prognosis of alcohol related hepatocellular carcinoma patients by establishing and validating a convenient, precise and specific nomogram.

To address this shortcoming, we employed a nomogram approach, a statistical model in predictive accuracy of a given outcome at an individual level. A study reported a nomogram prediction of individual prognosis of patients with HCC, primarily from viral hepatitis B, with good accuracy (27). Others have reported the nomogram for predicting survival in early stage HCC, unresectable HCC after hepatic arterial infusion chemotherapy, HCC with portal vein tumor thrombus after resection, and recurrence of HCC after surgery. Given an increase in the incidence of alcohol related hepatocellular carcinoma and the report that patients with alcohol related hepatocellular carcinoma had a markedly reduced overall survival, mainly because of a worse liver function and tumor characteristics at diagnosis, when compared to patients with non–alcohol-related HCC, a construction of a nomogram with a good predictive accuracy of patients with alcohol related hepatocellular carcinoma, is needed to tailor the treatment plans at the individual level.

Our model consists of 6 variables including serum pre-albumin, serum creatinine, mean corpuscular hemoglobin concentration, red cell distribution width, platelet counts, and TNM staging. Unlike serum albumin, the serum level of pre-albumin is less affected by the underlying liver disease; it is a well indicator for nutritional status with a shorter half-life than that of serum albumin (28, 29). The level of serum pre-albumin is adversely associated with the overall survival in patients with HCC post hepatic resection (30). A meta-analysis showed that low levels of pre-albumin were significantly associated with poor prognosis in patients with liver cancer (31). Serum creatinine, a byproduct of creatine metabolism in the muscle, is normally excreted in the urine (32). The baseline level of serum creatinine has been associated with the overall survival in patients with various cancers, and in our study, in patients with alcohol related hepatocellular carcinoma (33). One recent nomogram study (34) have shown that creatinine is an independent prognostic factor for alcohol-related liver disease, and our results further confirm that creatinine is also an independent prognostic factor in alcohol related hepatocellular carcinoma patients. Hematological parameters were identified as important predictors in patients with several advanced malignancies and hematological diseases (35). Mean corpuscular hemoglobin concentration is reported to be associated with prognosis in patients with HCC, especially after hepatectomy (36).Perhaps the different effect of MCHC on prognosis may due to the different etiologies of hepatocytes. Red cell distribution width (RDW) is an indicative of abnormal red blood cell survival and impaired erythropoiesis. Several studies reported the implications of RDW and outcomes in patients with cancer, however, the exact mechanism is not completely understood (37). The inflammatory process and the presence of oxidative stress secondary to cancer may contribute to the level of RDW. Patients with malignant tumor often have an elevated platelet counts (38). In a nested case-control study, an elevated platelet count was associated with increased risk of cancer at several sites (38). A large retrospective study (39) shows that lower PLT is associated with better outcomes in patients with advanced HCC. Furthermore, Hayashi T et al (40) reported that antiplatelet therapy significantly improves OS and reduces the risk of liver-related death in non-hepatitis B virus (HBV) and non-hepatitis C virus (HCV) HCC patients. Numerous prognostic studies (41, 42) show that TNM stage is an independent predictor of hepatocellular carcinoma prognosis. Besides, Tokushige et al (43) reported TNM stage was a risk factor for recurrence of alcohol related hepatocellular carcinoma based on a multicenter survey. Our study further elucidates TNM is an independent predictor of prognosis in alcohol related hepatocellular carcinoma patients.

By combining these variables into a nomogram, we are able to prognostically stratify alcohol related hepatocellular carcinoma patients based on the overall survival. The prognostic applicability of our nomogram was externally validated as shown in **Figures 4** and **5**. When compared our nomogram to the commonly used prognostic indicators such as the Child-Pugh and TNM staging, we found that our nomogram appears to have a better prognostic accuracy for overall survival (**Figure 5**). Of importance, our decision curve analysis also suggested that our nomogram illustrated a more net benefit than the Child-Pugh and TNM staging in predicting the 1-year, 3-year and 5-year survival (**Figure 6**).

The advantages of our study are a relatively large sample size with a longitudinal data on survival outcome. However, we acknowledge several limitations primarily due to the study design. Firstly, our alcohol related hepatocellular carcinoma patients were all male. And male and female responses to alcohol dehydrogenase activity may differ. Secondly, because this study was a single-center retrospective study, external cohort validation was not possible. Our nomogram was derived based on a single institute data with a homogenous cohort of patients with alcohol related hepatocellular carcinoma. Despite the fact that our nomogram was validated, it is based the similar patient characteristics. Morover, our hospital was the largest alcohol related hepatocellular carcinoma diagnosis and treatment hospital in China, with patients from all over the country every year. Internal validation shows good performance. Finally, unknown factors may influence OS in patients with alcohol related hepatocellular carcinoma. More data are needed to confirm the validity of this nomogram approach. Future studies to determine the prognostic accuracy in other cohorts or in Western patients are needed. To this end, a multicenter prospective study is underway.

In conclusion, we proposed the new nomogram based on readily available routine laboratory data in combination with TNM staging, as a model in predicting survival of patients with alcohol related hepatocellular carcinoma.

## Conclusion

The nomogram model we constructed can clearly and conveniently reflect the survival risk of alcohol related hepatocellular carcinoma patients. It can assist clinicians in judging the prognosis and treatment effect, which is conducive to the realization of precise and individualized treatment of alcohol related hepatocellular carcinoma patients.

## Data availability statement

The original contributions presented in the study are included in the article/**Supplementary Material**. Further inquiries can be directed to the corresponding author.

## Author contributions

CH and TY designed the initial research and wrote the article; JL and QG performed searches. GS, TW and CP extracted data. XJ and FY assessed the data quality, conducted statistical analyses. YLi, TK and JC assisted with statistical analyses and help reviewing the manuscript. LZ provided input into study design, analyze and reviewed the manuscript. YLu and SL supervised the research design, analysis and interpretation, and reviewed and castigated the manuscript. All authors contributed to the article and approved the submitted version.

## Funding

Our study was supported by the project: Study on the techniques for comprehensive early diagnosis of liver cancer with clinical application. SProgram name: Special program of sustainable development. Grant support: Issued by the Science, Technology and Innovation Commission of Shenzhen Municipality. Grant No.: KCXFZ202002011006448.

## Conflict of interest

The authors declare that the research was conducted in the absence of any commercial or financial relationships that could be construed as a potential conflict of interest.

## Publisher’s note

All claims expressed in this article are solely those of the authors and do not necessarily represent those of their affiliated organizations, or those of the publisher, the editors and the reviewers. Any product that may be evaluated in this article, or claim that may be made by its manufacturer, is not guaranteed or endorsed by the publisher.

## Supplementary material

Supplementary Figure 1 Kaplan–Meier curves of OS of patients in the training cohort and validation cohort.

Supplementary Figure 2 Kaplan–Meier survival curve stratified by a respective cut-off value for each variable in alcohol related hepatocellular carcinoma patients.

Supplementary Table 1 Treatment of alcohol related hepatocellular carcinoma patients during follow-up.

## Abbreviations

HCC, hepatocellular carcinoma, OS, overall survival, AUC, area under the curve, DCA, Decision curve analysis, TNM, tumor, node, metastasis, RDW, blood cell distribution width.
