# Supplementary figures and images for: Development and Validation of a nomogram for forecasting survival of alcohol related hepatocellular carcinoma patients
Source: Front Oncol. 2022 Nov 11;12:976445. doi: 10.3389/fonc.2022.976445 (PMC9692070; doi:10.3389/fonc.2022.976445)

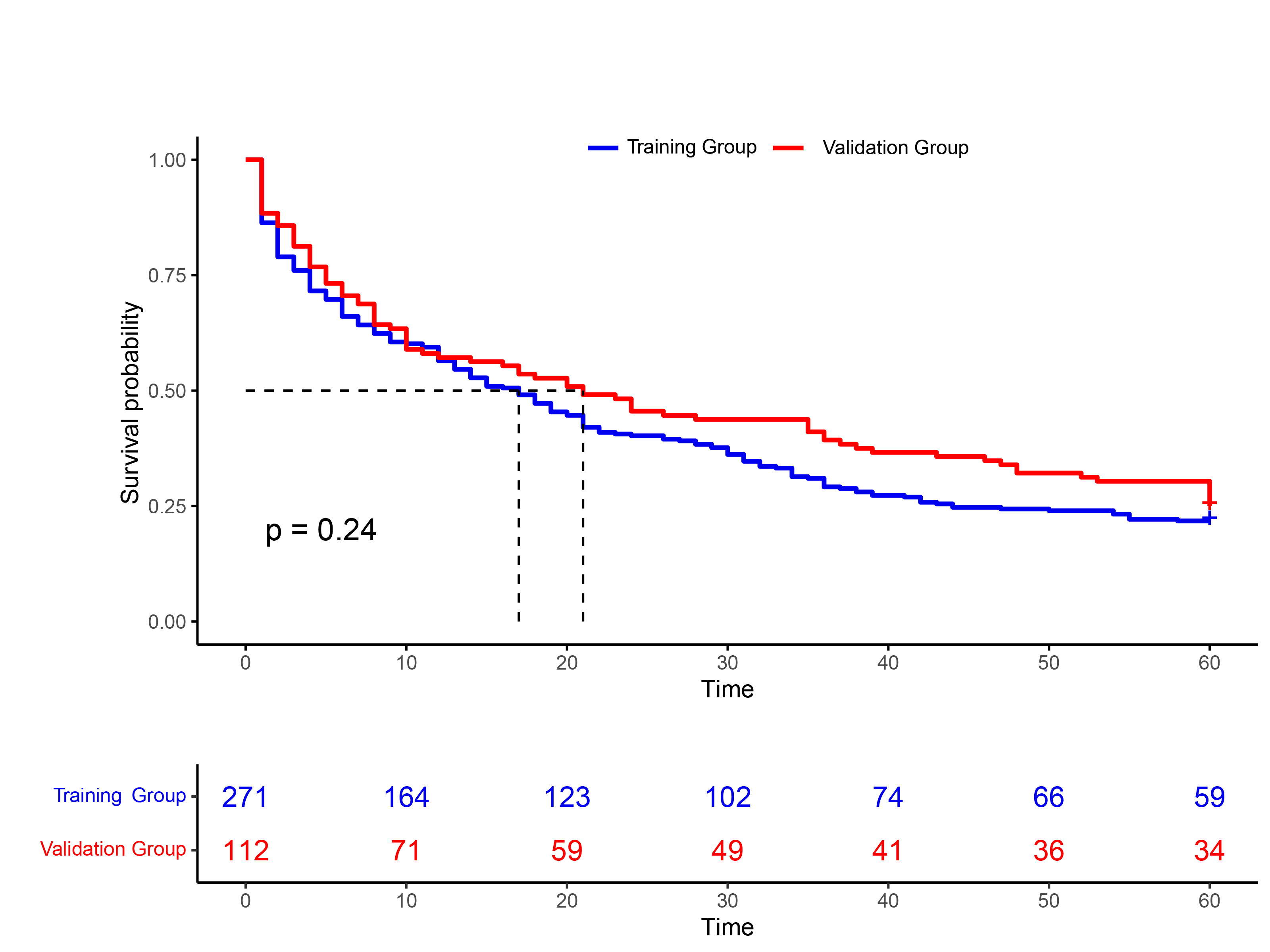

Supplement: Supplementary Figure 1 — Kaplan–Meier curves of OS of patients in the training cohort and validation cohort. [file Image_1.tif]

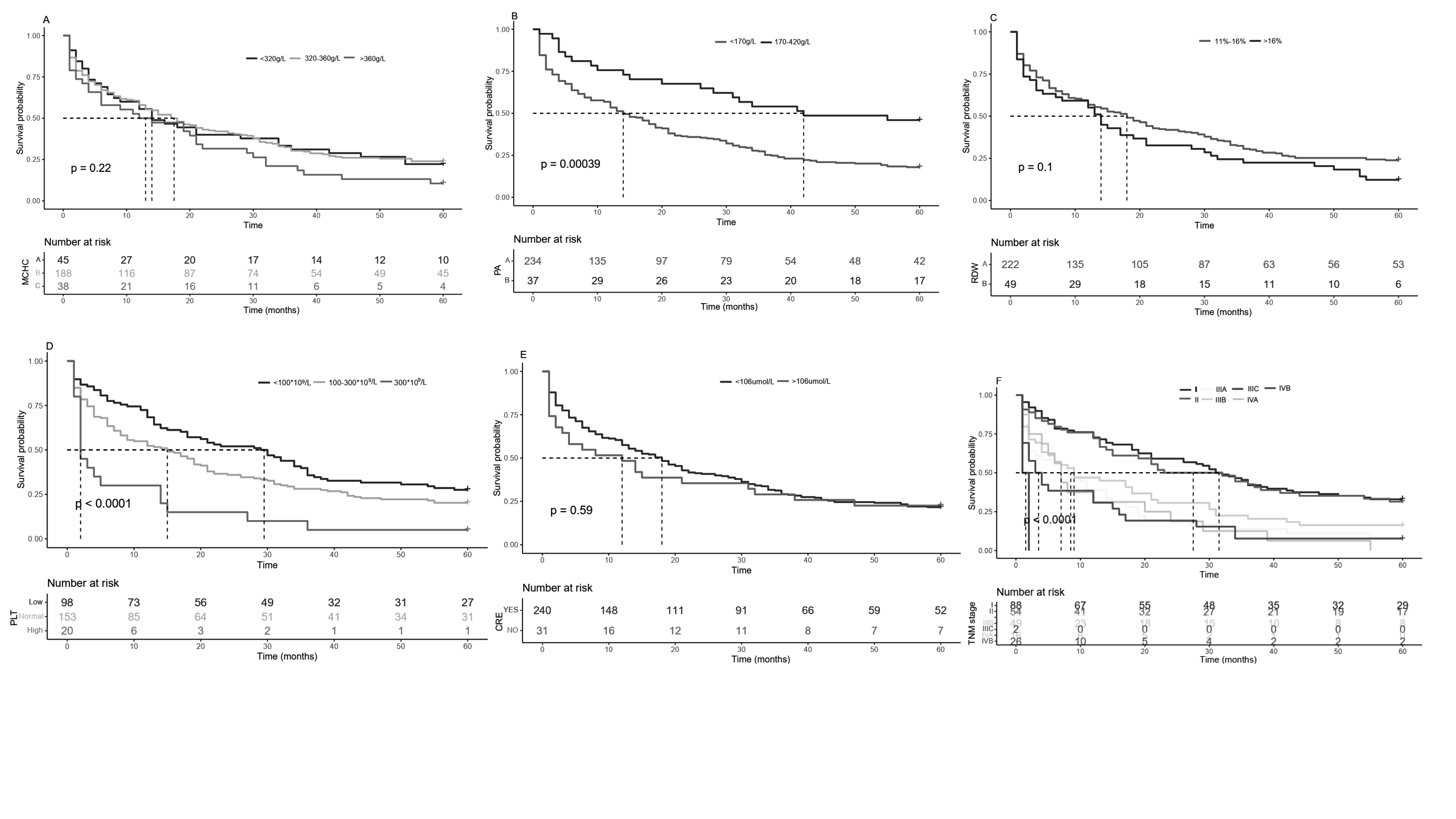

Supplement: Supplementary Figure 2 — Kaplan–Meier survival curve stratified by a respective cut-off value for each variable in alcohol related hepatocellular carcinoma patients. [file Image_2.tif]
