# Supplementary material for: Development and Validation of a nomogram for forecasting survival of alcohol related hepatocellular carcinoma patients
Source: Front Oncol. 2022 Nov 11;12:976445. doi: 10.3389/fonc.2022.976445 (PMC9692070; doi:10.3389/fonc.2022.976445)
Supplement: Supplementary Table 1 — Treatment of alcohol related hepatocellular carcinoma patients during follow-up. [file Table_1.docx]

| **Table S1: Treatment of AHC patients during follow-up** | | | | |
| --- | --- | --- | --- | --- |
|  | Treatment Options | Training Cohort (n=196) | Validation Cohort (n=96) |  |
|  | Surgery | 18 | 11 |  |
|  | Local ablation therapy | 6 | 3 |  |
|  | Intervention | 33 | 18 |  |
|  | Radiotherapy | 11 | 5 |  |
|  | Molecular Targeted Therapy | 25 | 11 |  |
|  | Systemic chemotherapy | 23 | 17 |  |
|  | Comprehensive Treatment | 80 | 31 |  |
